# Azathioprine as maintenance therapy for IgG4-related diseases: a retrospective case series and case-based review of the literature

**DOI:** 10.1007/s00296-026-06083-7

**Published:** 2026-02-14

**Authors:** Myriam Reisch, Walter Johann Spindelböck, Isabel Hodl, Daniel Pietsch, Florian Rainer, David Kickinger, Anamarija Sutic, Jens Thiel, Martin Stradner

**Affiliations:** 1 Department of Rheumatology and Immunology, Medical University of Graz, Graz, Austria; 2 Department of Gastroenterology and Hepatology, Medical University of Graz, Graz, Austria

**Keywords:** Immunoglobulin G4-related disease, Retrospective studies, Azathioprine, Antirheumatic agents, Recurrence

## Abstract

IgG4-related disease (IgG4-RD) is a rare fibro-inflammatory disorder characterized by progressive fibrosis involving multiple organs and tissues. Clinical manifestations are highly variable, reflecting the organs affected. Oral glucocorticoids (GC) are established as first-line therapy; however, relapse occurs in 15–60% of patients. To reduce relapse risk and GC-related adverse effects, GC-sparing strategies using disease-modifying antirheumatic drugs (DMARDs) may be considered. This study aims to evaluate the efficacy and safety of azathioprine in the management of IgG4-RD and provides an overview of the current literature. This retrospective case series assesses disease activity under azathioprine therapy in patients with IgG4-RD treated at the Medical University of Graz between 2006 and 2024. Patients with a confirmed IgG4-RD diagnosis according to the 2019 ACR/EULAR criteria who received azathioprine following initial GC therapy were included. Ten male patients (mean age at disease onset: 59.4 years) with a mean follow-up of 75.2 months were analyzed. All patients fulfilled the 2019 ACR/EULAR classification criteria. Indications for initiating azathioprine included disease relapse on GC (*n* = 3), GC-related adverse effects (*n* = 3), and the intention to reduce GC exposure (*n* = 7). During azathioprine therapy, three patients (30%) experienced a total of five relapses. Seven patients receiving azathioprine remained in remission throughout the observation period. Adverse events included infections (*n* = 2), hematologic abnormalities (*n* = 4), and elevated liver enzymes (*n* = 1). At last follow-up, five patients (50%) remained on azathioprine, two (20%) had switched to rituximab, one of whom was in remission and off therapy, and three patients (30%) were no longer receiving immunosuppressive treatment (two due to hematologic abnormalities, one by patient request), with no further therapy initiated. Azathioprine represents an appropriate maintenance therapy for IgG4-RD. In cases with relapse under azathioprine, alternative DMARDs, such as CD19- or CD20-targeted therapies, may be more effective.

**Supplementary Information:**

The online version contains supplementary material available at 10.1007/s00296-026-06083-7.

## Introduction

IgG4-related disease (IgG4-RD) is a rare fibro-inflammatory disorder [1] with an incidence of approximately 0.78 to 1.39 per 100,000 person-years [2], predominantly affecting individuals between 50 and 70 years of age. In contrast to many other immune-mediated diseases, men are more frequently affected than women, with a male-to-female ratio of 2.2:1 [3, 4].

IgG4-RD is characterized by fibrotic and proliferative components and can manifest itself in various organs and may lead to progressive tissue damage [5, 6]. The most commonly affected sites are the pancreas (pancreatitis; 60%), salivary glands (sialadenitis; 34%), kidneys (tubulointestinal nephritis; 23%), lacrimal glands (dacroadenitis; 23%), and the aorta (periaortitis; 20%) [7]. Retroperitoneal fibrosis may also develop in patients with IgG4-RD, with reported prevalence ranging from 9.6% to 27% [8].Hematological abnormalities can occur, including polyclonal hypergammaglobulinemia with elevated IgG4 levels, increased C-reactive protein (CRP) and erythrocyte sedimentation rate (ESR), eosinophilia, and reduced complement factors C3 and C4 [9, 10].

Diagnosis is based on the integration of clinical, imaging, and histopathological findings. In 2019, the ACR/EULAR classification criteria were published by Wallace et al. [11], proposing a three-step classification with a specificity of 97.8% and a sensitivity of 82.0%. The first-line treatment consists of oral glucocorticoids (GC), while surgical resection of affected tissues is considered only when necessary [12]. However, relapse rates remain high, with recurrence reported in 34–53% of patients after tapering, potentially leading to destructive fibrosis and organ failure [13]. To minimize relapse risk and GC-associated toxicity, GC-sparing immunosuppressive therapies are employed.

Most recently, a randomized controlled trial of B-cell–directed therapy with inebilizumab in patients with IgG4-RD was published, demonstrating confirmed efficacy [14]. Other therapeutic options, including mycophenolate mofetil (MMF), leflunomide, methotrexate, and rituximab, have also been described as potential treatment modalities. However, not all agents are suitable for every patient due to contraindications, side effects, and high costs [15]. 

Azathioprine represents another potential therapeutic option for IgG4-RD. However, to date, no randomized controlled trials have been conducted. Existing evidence is limited to a few case reports and case series focusing on individual organ manifestations, such as pulmonary involvement and reported variable outcomes [16]. Other organ involvements, including renal disease, have not been specifically studied. Consequently, the overall evidence for azathioprine in IgG4-RD is limited, with insufficient data regarding its safety profile, optimal treatment duration, and organ-specific efficacy [14, 17–19]. 

The aim of this study is to explore whether azathioprine may be suitable as a maintenance therapy in IgG4-RD with a focus on its potential effectiveness and safety profile and to provide an overview of the current literature on this topic.

## Methods

### Study design and population

We conducted a retrospective cohort study to evaluate disease activity during azathioprine maintenance therapy in patients with IgG4-RD treated at the Medical University of Graz, Austria.

Patients with a confirmed IgG4-RD diagnosis according to the 2019 ACR/EULAR classification criteria who had received current or prior azathioprine therapy were eligible for inclusion. Eligible cases were identified through the institutional electronic medical records system (openMedocs) and documented in a standardized database. Patients were excluded if clinical data were insufficient, if the observation period was less than 9 months, or if they did not meet the 2019 ACR/EULAR classification criteria.

### Outcomes

The primary outcome was disease activity and safety during azathioprine therapy. Disease activity, relapse, and remission were primarily based on the clinical judgment of the treating physician, supported by laboratory values. Imaging was performed when necessary to clarify the state of the disease. A minimum interval of three months was applied to define relapse, distinguishing new events from ongoing disease activity.

Secondary outcomes comprised organ involvement, prior and subsequent immunosuppressive therapies, histopathological findings, laboratory parameters (total IgG, IgG4, CRP, ESR, C3c, C4), treatment-related adverse effects, and comorbidities where available.

### Data analysis

Statistical analyses were descriptive. Continuous variables are presented as mean ± standard deviation (SD) or median with interquartile range (IQR), depending on distribution. Categorical variables are summarized as absolute numbers and percentages. Time-to-event analyses were performed using Kaplan–Meier curves to assess time to first relapse. The study period covered 2006–2024, as electronic records of the Medical University of Graz have been available since 2006. Statistical analyses were conducted using IBM SPSS Statistics, version 29, and Microsoft Excel.

### Ethics approval

Due to the retrospective nature of the study, a patient informed consent was not obtained. The study was conducted in accordance with the Declaration of Helsinki and was approved by the Ethics Committee of the Medical University of Graz (Approval No. 36–112) on February 9, 2024.

Preliminary results of this study were previously presented as an abstract at the 53rd Congress of the German Society for Rheumatology (DGRH) 2025 and published as: M.Reisch et al., Azathioprine as maintenance therapy for IgG4-related diseases: a retrospective case series, DGRH, 18.09.2025.

### Search strategy for the narrative review

A comprehensive literature search was conducted across databases, including Medline/PubMed, Cochrane Library, Web of Science, covering publications up to January 2026. The search terms used were “Azathioprine,” “Immunoglobulin G4-Related Disease,” and “Therapy.” We included case reports, case series, observational studies, randomized controlled trials, and review articles that specifically addressed the use of azathioprine as a treatment in IgG4-RD. Articles without mention azathioprine therapy in this context were excluded.

## Results

**Fig. 1:**
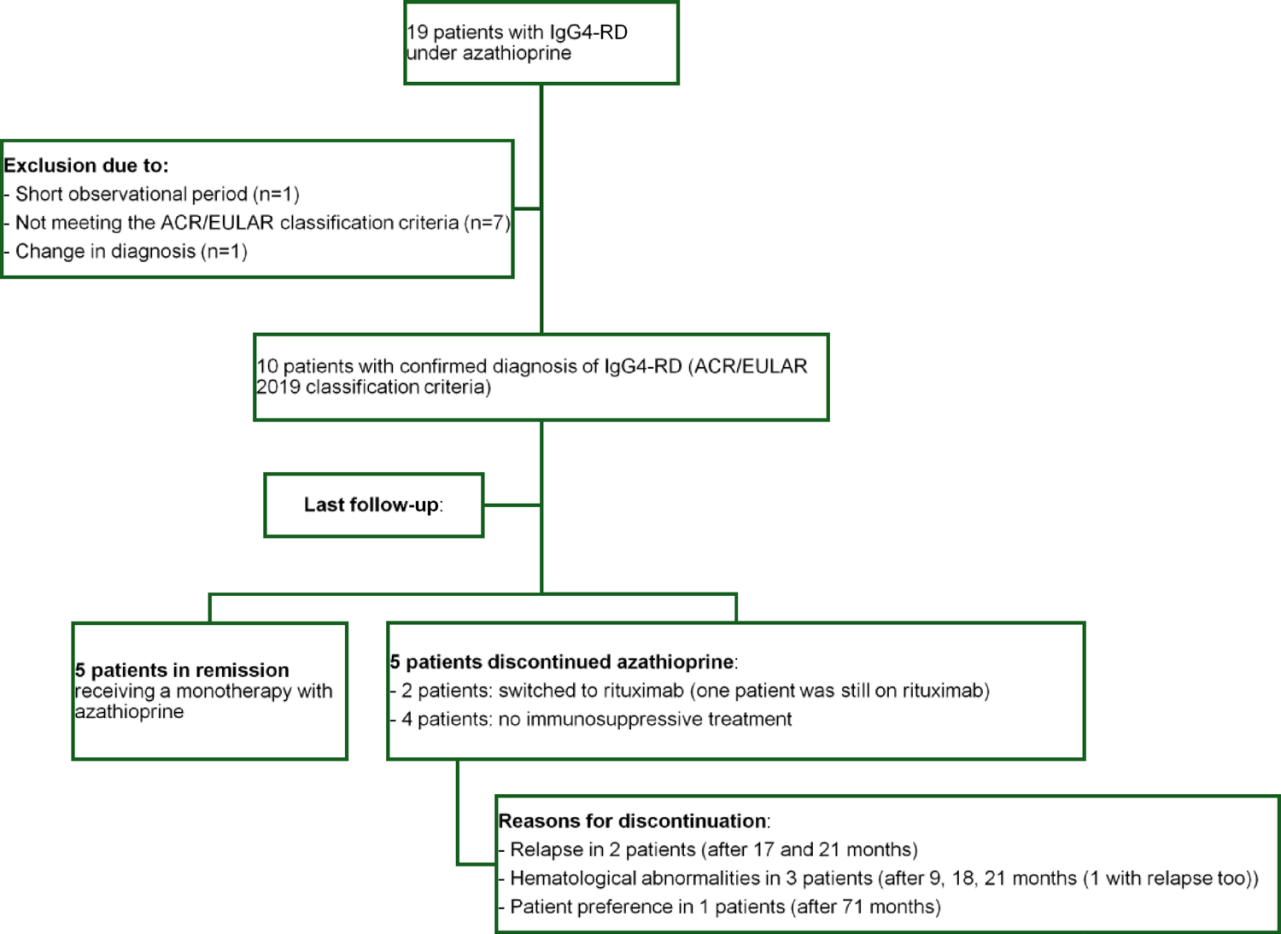
Flowchart: patients with Igg4-RD

A total of 19 patients with IgG4-RD treated with azathioprine were initially included. Of these, one patient was excluded due to a short observation period (< 9 months), seven patients did not meet the 2019 ACR/EULAR classification criteria, and one patient was excluded due to a change in diagnosis (Fig. 1).

A total of 10 patients were analyzed, all of whom were male (100%) and with a mean age at disease onset of 59.4 years (range: 32–78; SD: 13.69).

The mean follow-up period was 75.2 months (range: 16–171; SD: 48.65). Histopathological confirmation of the diagnosis was available in 9 out of 10 patients (90%). All patients (100%) fulfilled the ACR/EULAR 2019 classification criteria.

Organ involvement was heterogeneous, with the most commonly affected sites being the pancreas (*n* = 4), lymph nodes (*n* = 5), and retroperitoneal tissue (*n* = 3). Among the study cohort, six patients presented with involvement of a single organ, while four patients exhibited multisystem involvement affecting two or more organs (see Table 1). Organ damage was observed in two patients (20%), both of whom developed pancreatic insufficiency.

**Table 1:** Individual patient characteristics

Pat.-ID	Sex	Age at disease onset	Organ involvment	Therapy at last follow-up	Received therapy	Surgery	Reason for initiating azathioprine	Reason for stopping azathioprine	Relapse under azathioprine	Adverse events under azathioprine	Comorbidities
1	m	73	Parotid gland	Azathioprine	St.p. GC-therapy	Yes: Removal of the left parotid	Relapse under GC		No	Pneumonia (hospitalized)	Type 2 diabetes mellitus, arterial hypertension, psoriasis vulgaris, intraductal papillary mucinous tumors of the pancreas, chronic renal failure, atrioventricular nodal reentry tachycardia (St.p. electrophysiological ablation)
2	m	32	Pancreas, kidney (tubulointerstitial nephritis), lymphadenopathy	no treatment	St.p. GC-therapy, St.p. Azathioprine, St.p. Rituximab (50months)	No	Relapse under GC	Relapse	yes: 3x	No	Chronic renal failure, monoclonal gammopathy of undetermined significance, immune thrombocytopenia, St.p. splenectomy after splenic vein thrombosis
3	m	52	Pancreas	Azathioprine	St.p. GC-therapy	No	GC sparing		yes:1x	Anemia, increased liver enzymes	Type 2 diabetes mellitus, bronchial asthma, hypothyroidism
4	m	76	Pancreas, retroperitoneal fibrosis, lymphadenopathy	no treatment	St.p. GC-therapy, St.p. Azathioprine	No	GC side effect (infection) and GC sparing	Stopped due to leukopenia and anemia, no further therapy initiated	No	Leukopenia and anemia	Arterial hypertension
5	m	57	Retroperitoneal fibrosis	Azathioprine	St.p. GC-therapy	No	GC side effect (hyperglycemia, oedema)		No	No	Arterial hypertension, type 2 diabetes mellitus, St.p. deep venous thrombosis
6	m	52	Pancreas, kidney (tubulointerstitial nephritis), lymphadenopathy	Rituximab	St.p. GC-therapy, St.p. Azathioprine	No	GC side effect (psychiatric) and GC sparing	Relapse and aggravation of lymphopenia	Yes: 1x	Purulent pleuritis	Immunodeficiency after Rituximab administration, osteoporosis, St.p. vertebral fractures, depression, St.p. purulent pleuritis, St.p. urinary tract infection
7	m	58	Retroperitoneal fibrosis	Azathioprine	St.p. GC-therapy	No	GC sparing, 2x relapse under GC		No	No	Atopic eczema
8	m	56	Periaortitis	Azathioprine	St.p. GC-therapy	No	GC sparing		No	No	Chronic obstructive pulmonary disease, nicotine abusus, obesity
9	m	78	Parotid and submandibular glands, lymphadenopathia	No treatment	St.p. GC-therapy, St.p. Azathioprine	No	GC sparing and no remission under GC (prednisolone > 12.5 mg/d)	Pancytopenia, no further therapy initiated	No	Pancytopenia	Coronary heart disease stage III with a history of triple bypass surgery, St.p. pacemaker implantation, atrial fibrillation, arterial hypertension, hepatic steatosis
10	m	60	Cervical lymphadenopathy	no treatment	St.p. GC-therapy, St.p. Azathioprine	Yes: Radical cervical lymphadenectomy	GC sparing	At the patient’s request	no	Macrocytosis and leukopenia	Arterial hypertension, hyperlipidemia, type 2 diabetes mellitus, seborrheic dermatitis, recurrent perianal abscess

The median time from diagnosis to initiation of azathioprine therapy was 12.2 months (range: 3–29; SD: 9.20), with a mean treatment duration of 45.8 months (range: 9–159; SD: 43.88).

The main indications for initiating azathioprine therapy included disease relapse under GC (*n* = 3), GC-related adverse effects (*n* = 3), and the intention to reduce GC exposure (*n* = 7).

**Fig. 2:**
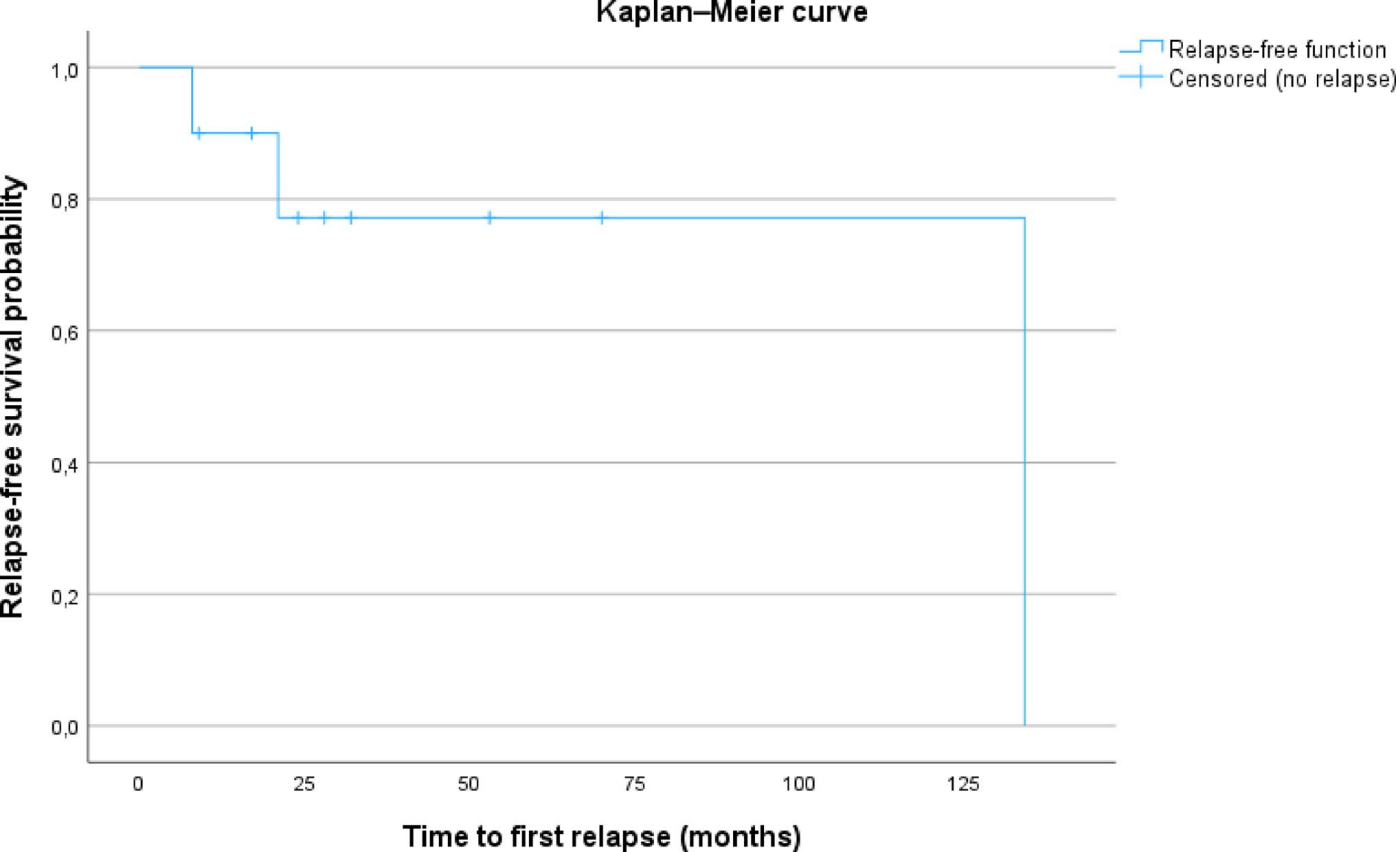
Time to first relapse under azathioprine in IgG4-RD. Kaplan–Meier curve showing time to first relapse in a cohort of 10 patients. The observation period extended from initiation of azathioprine therapy to the occurrence of the first relapse or last follow-up under azathioprine, ranging from 8 to 135 months. Relapses occurred in three patients (patient 2 at 8 months, patient 6 at 21 months, and patient 3 at 134 months). The remaining patients were relapse-free at last follow-up (patient 1: 24 months; patient 4: 9 months; patient 5: 32 months; patient 7: 53 months; patient 8: 28 months; patient 9: 17 months; patient 10: 70 months)

Disease relapse under azathioprine therapy occurred in three patients (30%; Patient 2, 3 and 6), with a total of five relapse episodes (see Fig. 2).

The average dose of azathioprine at therapy start was 175 mg/day (range: 100–300 mg, 0,92 − 2,27 mg/kg/d). Patients who experienced a relapse received a mean azathioprine dose of 167 mg/d (range 150–200 mg; mean: 2.08 mg/kg/d), whereas patients without relapse were treated with a mean dose of 179 mg/d (range 100–300 mg; mean: 1.7 mg/kg/d). Overall, weight-adjusted azathioprine doses were lower on average in patients who remained in remission compared with those who experienced relapse.

At the last follow-up, five patients (50%) were still receiving a monotherapy with azathioprine, while two patients (20%) had been switched to rituximab after discontinuation of azathioprine. One patient discontinued rituximab therapy after achieving sustained remission. In total, four patients (40%) were no longer receiving any immunosuppressive treatment.

Discontinuation of azathioprine occurred for the following reasons: relapse in two patients (after 17 and 21 months of treatment), hematological abnormalities in three patients (after 9, 18 months and after 21 months (with relapse too) in one patient), and due to patient preference in one case (after 71 months). In three cases, no further immunosuppressive therapy was initiated.

Notably, all patients with renal involvement (*n* = 2) experienced disease relapse under azathioprine therapy. At treatment initiation, these patients also exhibited elevated IgG4 and total IgG levels, as well as increased CRP levels. Multimorbidity did not appear to be descriptively different or more pronounced compared with patients without renal involvement. Complement levels (C3 and C4) were unremarkable in one patient, while only a single normal measurement was available for the other, precluding meaningful conclusions regarding complement consumption.

### Safety of azathioprine

Reported adverse events associated with a monotherapy azathioprine included infections (*n* = 2), hematological abnormalities (*n* = 4), and elevated liver enzymes (*n* = 1) (see Table 2). Hematological abnormalities included leukopenia with anemia, pancytopenia, isolated anemia, and macrocytosis with leukopenia, each occurring in one patient.

**Table 2:** Characteristics of the patients with IgG4-RD (*n* = 10)

Sex	Male 10/10
Age at onset (in years)	59.4* (32–78; SD 13.69)
Follow-up time (in months)	75.2* (16–171; SD 48.65)
ACR/EULAR 2019 classification criteria fulfilled	10/10
Biopsy available	09/10
Organ involvement	Parotid gland 1/10, submandibular gland 1/10, pancreas 4/10, kidney 2/10, retroperitoneal fibrosis 3/10, lymphadenopathy 5/10, periaortitis 1/10
Number of affected organs per patient	1.7* (1–3)
Organ damage	Pancreatic insufficiency: 2/10, renal insufficiency: 1/10
Therapy at last follow-up	Azathioprine: 5/10, rituximab after azathioprine: 1/10, no therapy: 4/10
Time from diagnosis to azathioprine initiation (months)	12.2* (3–29; SD 9.20)
Azathioprine treatment duration (months)	45.8* (9–159; SD43.88)
Previous therapies (excluding azathioprine)	Glucocorticoid 10/10, rituximab 1/10
Surgical resection of affected tissues	02/10
Main indication for initiation of azathioprine	Relapse (3x), glucocorticoid side effects (3x), glucocorticoid sparing (7x)
Reason for stopping azathioprine	Relapse (2x), alterations of blood count (3x), patient preference (1x
Relapse under azathioprine	03/10
Adverse events under a monotherapy with azathioprine	Infection (2x), alterations of blood count (4x), increased liver enzymes (1x)

*SD* standard deviation, * mean

### Laboratory analyses

Serum biomarkers such as IgG4, total IgG, CRP, ESR, and complement factors (C3c, C4) were analyzed in a subset of patients. Overall, IgG4 and total IgG concentrations were heterogeneous, with persistently elevated concentrations observed in patients who subsequently relapsed under azathioprine therapy. In contrast, patients whose IgG4 concentrations were within the normal range at baseline or normalized during follow-up remained in stable remission (see Tables S1, S2). Elevated CRP concentrations at treatment initiation were predominantly seen in patients who later relapsed, suggesting that CRP may serve as a potential risk marker for relapses (see Table S3). ESR values generally declined during therapy irrespective of relapse status (see Table S4). Regarding complement, C3c and C4 consumption was only evident in a single patient who relapsed, while other patients maintained normal complement concentrations under stable azathioprine maintenance (see Tables S5, S6).

## Discussion

Patients with IgG4-RD often experience relapse when GC monotherapy is tapered or withdrawn. To reduce both relapse rates and cumulative GC exposure, additional immunosuppressants such as azathioprine are recommended [13].

In this study, azathioprine was evaluated as maintenance therapy for IgG4-RD following GC induction. Azathioprine may represent a suitable maintenance option; however, in our small cohort, three patients experienced a total of five relapses during therapy. The safety profile was favorable, with reported adverse events including two infections, four cases of hematologic abnormalities, and one case of elevated liver enzymes. Three patients had to discontinue therapy because of these hematologic abnormalities.

High relapse rates have been reported in IgG4-RD, with both recurrent involvement of previously affected organs and de novo organ manifestations during follow-up. In large cohort studies and a recent meta-analysis by Yang et al. [20] (24 studies, 3,797 patients), cumulative relapse rates were approximately 17% at 12 months, 26% at 24 months, and 33% at 36 months after initial treatment. After GC discontinuation, relapse rates of up to 53% have been reported in patients [13].

Several factors influence relapse risk. The number of organs involved is an independent predictor, with each additional organ increasing the likelihood of relapse [20]. Elevated baseline or persistently high serum IgG4 levels, as well as complement consumption (low C3/C4), have been associated with higher relapse risk. Additional factors include a history of allergy, baseline eosinophilia, and re-elevation of serum IgG4 during follow-up. Withdrawal or tapering of GC is a strong independent predictor of relapse, as confirmed in multiple studies [20–22]. In our cohort, patients who relapsed on azathioprine therapy showed persistently elevated IgG4 and total IgG levels, whereas those with normal or normalized IgG4 values remained in stable remission.

In the literature, patients with IgG4-RD who present with hypocomplementemia are described as having a more active clinical phenotype of the disease [23]. In our study complement consumption (C3c, C4) was observed in one relapsing patient. All patients in remission maintained normal complement levels during azathioprine treatment.

Elevated CRP and ESR have also been identified as risk factors for disease relapse [22]. In our cohort elevated CRP at treatment initiation was mainly observed in patients who later relapsed, indicating its potential as a relapse risk marker, whereas ESR declined during therapy regardless of relapse status. Accordingly, our findings suggest that elevated CRP and IgG4 levels may be associated with an increased risk of relapse, even in patients receiving azathioprine therapy.

In 2023, European expert-based consensus statements on the management of IgG4-RD were published [24]. GC are recommended as first-line therapy and should be tapered progressively. Maintenance therapy is advised for patients with high disease activity or relapse risk, using GC-sparing agents, including biologics or conventional DMARDs such as azathioprine, mycophenolate mofetil, leflunomid and methotrexate. Rituximab can be used for both induction and maintenance therapy.

However, no randomized controlled trials specifically evaluating azathioprine in IgG4-RD have been published to date. Current evidence is limited. Small case reports demonstrate efficacy, primarily supporting its use in combination with GC to reduce relapse risk and minimize steroid exposure [25–28].

Moreover, only a few observational studies with small patient numbers are available, showing inconsistent findings, and in several studies azathioprine was not evaluated as an isolated therapy:

In a Spanish multicenter cohort of 68 patients with IgG4-RD, 13 patients received azathioprine [29]. All achieved partial or complete response (100%; complete response: 45.5 ± 52.2; relapse: 16.7 ± 38.9; treatment failure: 16.7 ± 38.9). In comparison, patients treated with mycophenolate mofetil (*n* = 6) also demonstrated universal partial/complete response (100%), with a complete response rate of 80.0 ± 44.7, and no relapses or failures. Overall, azathioprine plus GC appeared more likely to achieve partial or complete response compared with surgery alone (OR 3 [0.2–46]).

In a cohort of 53 patients with IgG4-associated cholangitis (IAC), only four patients were treated with azathioprine, of whom one relapsed while on a low-dose of 50 mg/d. In this patient, remission was achieved following an increase in the dose to 2–2.5.5 mg/kg, with no further relapses. All four patients maintained remission on immunomodulatory therapy (median follow-up: 6 months; range, 2–19 months). Based on these findings, the authors suggest an azathioprine dose of 2–2.5.5 mg/kg [30]. However, in our study, weight-adjusted azathioprine doses were lower on average in patients who remained in remission (mean: 1.7 mg/kg/d) compared with those who experienced relapse (mean: 2.08 mg/kg/d).

In a systematic literature review, therapeutic efficacy was demonstrated in 56 of 69 relapses (81%) treated with azathioprine, which was initiated following disease relapse. However, no further analyses specifically evaluating the effectiveness of azathioprine were conducted [31].

In a systematic review of 169 patients with IgG4-related pachymeningitis, azathioprine was administered in 21 cases (12%) [32]. Although relapses were reported during azathioprine treatment, its efficacy was not evaluated independently but rather alongside other immunosuppressants. Notably, azathioprine’s role remains unclear due to the lack of isolated analysis. In contrast, rituximab demonstrated superior efficacy, with a significantly lower rate of refractory disease (3/31; 9.7%) compared to other immunosuppressive agents (11/36; 31%, *P* = 0.03) in patients with neurological or systemic biopsy-confirmed IgG4-RD.

In contrast to the studies outlined above, a case series of 15 patients with IgG4–related lung disease treated with GC and azathioprine (median treatment duration: 14 months) was evaluated using serial imaging over a median follow-up interval of 12 months. Complete remission was observed only in 2 patients (6.5%), partial remission in 22 (71.0%), and stable disease in 7 (22.6%). Among patients with stable disease as the initial response, two experienced disease progression 10 and 11 months after treatment discontinuation, one achieved partial remission, and one remained stable [16].

In another study including 12 patients treated with azathioprine, only one patient (8%) achieved a complete response [32]. This patient had pancreatic and lymph node involvement, and azathioprine was successfully continued as maintenance therapy following GC induction. Azathioprine was discontinued in 6 of 12 patients (50%) due to toxicity, including hepatic, gastrointestinal, and muscular adverse effects, before treatment efficacy could be adequately assessed. In the remaining five patients, azathioprine failed to sufficiently control disease activity despite treatment durations exceeding three months, leading to discontinuation.

Overall, there is no strong evidence regarding the efficacy of azathioprine across different patterns of organ involvement, whether in single- or multi-organ disease. This study directly provides data on 10 patients with IgG4-RD meeting ACR/EULAR classification criteria, demonstrating an overall favorable response as maintenance therapy and suggesting a potential alternative DMARD strategy. However, the absence of randomized controlled trials and the heterogeneity of outcomes preclude meaningful comparative analyses for azathioprine in IgG4-RD.

Recent randomized controlled and open-label trials have demonstrated encouraging efficacy signals for several alternative GC-sparing agents beyond azathioprine.

In a 12-month, open-label trial, patients with active IgG4-RD were randomized to leflunomide plus GC versus GC monotherapy. Combination therapy was significantly more effective in prolonging the time to relapse compared with monotherapy (HR 0.35, 95% CI 0.13–0.90, *P* = 0.023) [33].

Another open-label randomized controlled trial included 94 patients with newly diagnosed IgG4-RD, comparing MMF (1–1.5 g/day) plus GC with GC monotherapy. After 12 months, the complete response rate was significantly higher in the MMF + GC group, and the cumulative relapse rate was markedly lower (20.6% vs. 40.0%) [34].

Rituximab, a B-cell depleting therapy, has consistently demonstrated efficacy in IgG4-RD across multiple cohort studies, prospective trials, and meta-analyses [35–37]. In a prospective open-label trial, Carruthers et al. [38] treated 30 patients with two doses of rituximab (1000 mg each) without concomitant GC or with successful GC tapering within two months. The primary outcome (IgG4-RD responder index decline ≥ 2 points from baseline, absence of flares before month 6, and no GC use between months 2 and 6) was achieved by 23 patients (77%). At 6 months, 47% were in complete remission, and 40% maintained complete remission at 12 months.

However, another prospective study reported that patients receiving only a single course of rituximab had a relapse rate of 71% after 18 months, compared with no relapses in those maintained on rituximab for 6 months [36]. These findings suggest that while rituximab can induce remission even without GC, maintenance therapy is necessary to prevent disease recurrence.

A phase 3, multicenter, double-blind, randomized, placebo-controlled trial evaluated inebilizumab, a CD19-targeted B-cell-depleting therapy, in adults with active IgG4-RD [14]. A total of 135 patients were randomized, with 68 receiving inebilizumab and 67 receiving placebo. Treatment with inebilizumab significantly reduced the risk of disease flare: Seven patients (10%) in the inebilizumab group experienced at least one flare compared with 40 patients (60%) in the placebo group (HR 0.13, 95% CI 0.06–0.28, *P* < 0.001). These findings highlight the efficacy of CD19-directed B-cell depletion in reducing flares and promoting sustained remission in IgG4-RD.

Beyond B-cell-directed therapies, several case reports and small series investigating abatacept, belimumab, dupilumab, tocilizumab, and januskinase (JAK) inhibitors have also shown promising results, although robust evidence is still lacking [39–48].

This study provides valuable real-world data on azathioprine use in patients with IgG4-RD across various organ involvements, all classified by ACR/EULAR criteria 2019. Given the limited existing research on azathioprine as maintenance therapy in IgG4-RD, our findings add information on safety and risk of relapse of azathioprine. Overall, several limitations should be acknowledged. First, the study has a retrospective design. Due to the retrospective design of this study, some data were incomplete or unavailable, which may introduce bias and limit the comprehensiveness of the analysis. Certain clinical or laboratory parameters were not consistently documented. Second, the sample size is small due to the rarity of the disease; furthermore, the strict application of the 2019 ACR/EULAR classification criteria allowed inclusion of only 10 patients from our cohort, although initially 19 patients were considered for inclusion. This reduction may increase the risk of selection bias. Third, the single-center study design limits the generalizability of the findings. Finally, no direct control group was available for comparison with the azathioprine-treated patients, precluding conclusions regarding the effects of different therapeutic strategies.

In this case series, azathioprine may have been less effective in patients with IgG4-RD and renal involvement, however, due to the small number of case, definite conclusion cannot be drawn. Although this observation is preliminary and therefore not generalizable, it is hypothesis-generating and raises the possibility that a stratified approach based on organ involvement and risk profile could be considered in IgG4-RD. Patients could potentially be categorized according to the severity of organ involvement (life-threatening versus mild, non-organ-threatening disease) and therapy could be adapted accordingly. Specifically, in cases of severe organ involvement (e.g., kidney, lung), direct therapy with B-cell-targeted agents may be required to achieve remission, whereas azathioprine might be adequate in patients with mild, non-organ-threatening manifestations.

## Conclusion

Azathioprine may be a potentially effective and well-tolerated option for maintenance therapy in patients with IgG4-RD, with a favorable safety profile observed in our cohort. In the majority of patients, azathioprine was associated with stable remission without the need for additional GC therapy and might contribute to a reduced risk of relapse. Nevertheless, disease recurrence occurred in three patients. Treatment with rituximab subsequently induced remission in these cases. These findings suggest that, particularly in patients with IgG4-RD and relapse under azathioprine, alternative DMARDs such as rituximab or inebilizumab could be more effective. Larger, randomized studies are needed to confirm these observations and to better define optimal maintenance strategies in IgG4-RD.

## Supplementary Information

Below is the link to the electronic supplementary material.

Supplementary file1 (DOCX 26395 KB)

## Data Availability

The authors confirm that the data supporting the findings of this study are available within the article and its supplementary materials.
