# Supplementary material for: Azathioprine as maintenance therapy for IgG4-related diseases: a retrospective case series and case-based review of the literature
Source: Rheumatol Int. 2026 Feb 14;46(3):47. doi: 10.1007/s00296-026-06083-7 (PMC12906543; doi:10.1007/s00296-026-06083-7)
Supplement: Supplementary file 1 — Supplementary file1 (DOCX 26395 KB) [file 296_2026_6083_MOESM1_ESM.docx]

**Supplementary labarotory results**

The number of study participants was insufficient to reliably assess serum IgG4, total IgG, CRP, ESR, C3c, and C4 levels as longitudinal biomarkers for predicting or determining disease activity. Nevertheless, some trends could be observed and are summarized below.

**IgG4 in our patient cohort**

IgG4 levels among patients were highly heterogeneous. Notably, in patients 2 and 6, both of whom experienced disease relapse, markedly elevated IgG4 concentrations were documented. These values did not decline under azathioprine therapy but rather exhibited a fluctuating course with a tendency toward further elevation. By contrast, patients with IgG4 levels within the normal range at baseline, or whose values normalized during azathioprine (AZA) therapy, remained in stable remission.

Table S1: Serum IgG4 concentrations (g/L) in the patient cohort

| Level of IgG4 in g/L (0.030-2.010) | Pat. 1 | Pat. 2 | Pat. 3 | Pat. 4 | Pat. 5 | Pat. 6 | Pat. 7 | Pat. 8 | Pat. 9 | Pat. 10 |
| --- | --- | --- | --- | --- | --- | --- | --- | --- | --- | --- |
| At diagnosis | 1.070 | 2.580 | Not measured | 5.410 | 0.307 | 12.400 | 2.710 | 2.390 | 44.000 | 0.960 |
| Start of AZA | Not measured | 15.800 | Not measured | 1.620 | 0.064 | 18.100 | 0.746 | 2.070 | 28.300 | 0.255 |
| 6 months of AZA | 0.664 | 14.800 | Not measured | 1.690 | 0.126 | 60.60 | 0.871 | 1.210 | 5.680 | 0.360 |
| 12 months of AZA | 0.821 | 26.200 | Not measured | Not measured | 0.111 | 15.100 | 1.530 | 1.430 | 3.750 | 0.491 |
| 24 months of AZA | 0.676 | Not measured | Not measured | Not measured | 0.153 | 24.400 | 1.040 | 1.410 | Not measured | 0.432 |
| 36 months of AZA | 0.617 | Not measured | Not measured | Not measured | 0.137 | Not measured | 1.130 | 1.510 | Not measured | 0.584 |

Figure S1: Temporal course of the serum IgG4 concentrations (g/L) in individual patients

**Total IgG in our patient cohort**

A similar pattern was observed for total IgG. Patients 2 and 6, both relapsing cases, showed persistently elevated IgG levels under azathioprine treatment. The remaining patients either had normal IgG values at baseline or achieved normalization during follow-up.

Table S2: Serum IgG concentrations (g/L) in the patient cohort

| Level of IgG in g/L (7.00-16.00) | Pat. 1 | Pat. 2 | Pat. 3 | Pat. 4 | Pat. 5 | Pat. 6 | Pat. 7 | Pat. 8 | Pat. 9 | Pat. 10 |
| --- | --- | --- | --- | --- | --- | --- | --- | --- | --- | --- |
| At diagnosis | 17.20 | 15.30 | Not measured | 24.10 | 12.30 | 22.30 | 15.80 | 16.20 | 44.10 | 14.60 |
| Start of AZA | 18.20 | 35.10 | Not measured | 10.40 | 6.94 | 25.90 | 12.50 | 15.50 | 26.80 | 10.30 |
| 6 months of AZA | 12.90 | 28.40 | Not measured | 12.90 | 8.51 | 54.60 | 12.80 | 11.50 | 14.30 | 12.20 |
| 12 months of AZA | 13.30 | 42.40 | Not measured | Not measured | 9.11 | 17.40 | 12.90 | 12.70 | 12.50 | 10.40 |
| 24 months of AZA | 16.50 | Not measured | Not measured | Not measured | 10.90 | 30.1 | 11.50 | 14.30 | Not measured | 11.50 |
| 36 months of AZA | 14.10 | Not measured | Not measured | Not measured | 10.40 | Not measured | 12.80 | 13.60 | Not measured | 11.60 |

Figure S2: Temporal course of the serum IgG concentrations (g/L) in individual patients

**C-reactive protein (CRP) in our patient cohort**

At the initiation of azathioprine therapy, patients who subsequently experienced relapse displayed markedly elevated CRP levels compared with those who remained relapse-free. This finding may suggest that CRP could serve as a potential risk factor for relapse.

Table S3: C-reactive protein concentrations (mg/L) in the patient cohort

| Level of CRP in mg/L (0-5.0) | Pat. 1 | Pat. 2 | Pat. 3 | Pat. 4 | Pat. 5 | Pat. 6 | Pat. 7 | Pat. 8 | Pat. 9 | Pat. 10 |
| --- | --- | --- | --- | --- | --- | --- | --- | --- | --- | --- |
| At diagnosis | 4.3 | 13.9 | Not measured | 7.9 | 3.5 | 20.5 | 47.1 | 4.8 | 1.4 | 5.5 |
| Start of AZA | 1.4 | 12.6 | Not measured | 27.1 | 3.3 | 25.9 | 6.0 | 1.1 | 0 | 4.2 |
| 6 months of AZA | 0.07 | 2.1 | Not measured | 6.0 | 4.5 | 6.8 | 3.9 | 2.6 | 1.0 | 0.6 |
| 12 months of AZA | 1.6 | 1.2 | Not measured | Not measured | 0.9 | 1.9 | 6.4 | 2.4 | 0.6 | 0 |
| 24 months of AZA | 29.1 | Not measured | Not measured | Not measured | 1.5 | 4.5 | 3.6 | 1.0 | Not measured | 0 |
| 36 months of AZA | 4 | Not measured | Not measured | Not measured | Not measured | Not measured | 3.0 | Not measured | Not measured | 0 |

Figure S3: Temporal course of the C-reactive protein (CRP) (mg/L) in individual patients

**Erythrocyte sedimentation rate (ESR) in our patient cohort**

Three of five patients presented with elevated ESR values at diagnosis. During treatment, ESR levels showed a decline, irrespective of relapse status.

Table S4: Erythrocyte sedimentation rate (ESR) (mm/h) in the patient cohort

| Level of ESR in mm/h (0-20) | Pat. 1 | Pat. 2 | Pat. 3 | Pat. 4 | Pat. 5 | Pat. 6 | Pat. 7 | Pat. 8 | Pat. 9 | Pat. 10 |
| --- | --- | --- | --- | --- | --- | --- | --- | --- | --- | --- |
| At diagnosis | Not measured | Not measured | Not measured | 91 | Not measured | Not measured | 30 | 10 | 76 | 19 |
| Start of AZA | Not measured | 44 | Not measured | 88 | Not measured | Not measured | 14 | 6 | 29 | 11 |
| 6 months of AZA | 14 | 7 | Not measured | 58 | 14 | 99 | 16 | 4 | Not measured | 14 |
| 12 months of AZA | 16 | 14 | Not measured | Not measured | 12 | Not measured | 18 | Not measured | Not measured | 13 |
| 24 months of AZA | 49 | Not measured | Not measured | Not measured | 20 | Not measured | 13 | Not measured | Not measured | 9 |
| 36 months of AZA | 44 | Not measured | Not measured | Not measured | Not measured | Not measured | 16 | Not measured | Not measured | Not measured |

Figure S4: Temporal course of the Erythrocyte sedimentation rate (ESR) (mm/h) in individual patients in the patient cohort

**Complement C3c in our patient cohort**

In patients with a favorable response to azathioprine, no evidence of C3c consumption was observed during follow-up, with the exception of patient 2, who subsequently developed a relapse. Complement data were unavailable for the other relapsing patients, precluding firm conclusions regarding the role of complement consumption in relation to disease activity in IgG4-RD.

Table S5: Complement C3c (g/L) in the patient cohort

| Level of Complement C3c in g/L (0.900-1.800) | Pat. 1 | Pat. 2 | Pat. 3 | Pat. 4 | Pat. 5 | Pat. 6 | Pat. 7 | Pat. 8 | Pat. 9 | Pat. 10 |
| --- | --- | --- | --- | --- | --- | --- | --- | --- | --- | --- |
| At diagnosis | 0.797 | Not measured | Not measured | 0.956 | 1.420 | Not measured | 1.670 | Not measured | 0.428 | 1.700 |
| Start of AZA | 0.672 | 0.236 | Not measured | Not measured | 1.660 | Not measured | 1.180 | 1.130 | 0.777 | 1.300 |
| 6 months of AZA | 1.010 | 0.380 | Not measured | Not measured | 1.420 | Not measured | 1.260 | Not measured | 0.920 | 1.130 |
| 12 months of AZA | 1.150 | 0.221 | Not measured | Not measured | 1.260 | Not measured | 1.100 | Not measured | 0.913 | 1.250 |
| 24 months of AZA | 1.310 | Not measured | Not measured | Not measured | 1.520 | 0.746 | 0.982 | Not measured | Not measured | 1.090 |
| 36 months of AZA | 1.350 | Not measured | Not measured | Not measured | 1.530 | Not measured | 1.180 | Not measured | Not measured | 1.140 |

**Complement C4 in our patient cohort**

Only patient 2 demonstrated marked C4 consumption. Complement data were not available for patients 3 and 6, both of whom experienced relapse. Apart from these cases, patients receiving stable maintenance therapy with azathioprine consistently exhibited normal C4 values.

Table S6: Complement C4 (g/L) in the patient cohort

| Level of compliment C4 in g/L (0.100-0.400) | Pat. 1 | Pat. 2 | Pat. 3 | Pat. 4 | Pat. 5 | Pat. 6 | Pat. 7 | Pat. 8 | Pat. 9 | Pat. 10 |
| --- | --- | --- | --- | --- | --- | --- | --- | --- | --- | --- |
| At diagnosis | 0.068 | Not measured | Not measured | 0.129 | 0.345 | Not measured | 0.361 | Not measured | 0.020 | 0.398 |
| Start of AZA | 0.03 | 0.017 | Not measured | Not measured | 0.335 | Not measured | 0.256 | 0.197 | 0.088 | 0.361 |
| 6 months of AZA | 0.206 | 0 | Not measured | Not measured | 0.227 | Not measured | 0.277 | Not measured | 0.158 | 0.244 |
| 12 months of AZA | 0.264 | 0 | Not measured | Not measured | 0.182 | Not measured | 0.252 | Not measured | 0.161 | 0.246 |
| 24 months of AZA | 0.331 | Not measured | Not measured | Not measured | 0.303 | 0.093 | 0.220 | 0.242 | Not measured | 0.245 |
| 36 months of AZA | 0.355 | Not measured | Not measured | Not measured | 0.313 | Not measured | 0.268 | 0.264 | Not measured | 0.254 |

Legend:

AZA = Azathioprine

CRP = C-reactive protein

ESR = Erythrocyte sedimentation rate

C3c = Complement 3c

C4 = Complement 4
